# Clinical efficacy of greater trochanter osteotomy with tension wire fixation in total hip arthroplasty for Crowe type IV developmental dysplasia of the hip

**DOI:** 10.1186/s13018-023-04344-w

**Published:** 2024-01-03

**Authors:** Jinhui Peng, Ziye Liu, Zheru Ding, Qirong Qian, Yuli Wu

**Affiliations:** Department of Orthopedics, Shanghai Changzheng Hospital, Naval Medical University, No.415, Fengyang Road, Huangpu District, Shanghai, 200003 China

**Keywords:** High hip dislocation, Hip dysplasia reconstruction, Osteotomy, Total hip arthroplasty

## Abstract

**Objective:**

The choice of osteotomy in joint replacement surgery for Crowe type IV developmental dysplasia of the hip (DDH) is a challenging and controversial procedure. In this study, we compared the clinical efficacy of a combination of greater trochanter osteotomy and tension wire fixation with that of subtrochanteric osteotomy.

**Methods:**

We performed 15 primary total hip arthroplasty (THA) procedures between January 2016 and July 2020 on 13 patients with a combination of greater trochanter osteotomy and tension wire fixation (the GTT group) and 12 THA procedures in 11 patients using subtrochanteric osteotomy (the STO group). The mean follow-up was 2.8 years (range 2.2–4.5 years) in the GTT group and 2.6 years (range 2.5–4.3 years) in the STO group. Clinical scores and radiographic results were evaluated during the final follow-up for the 15 hips in the GTT group and 12 hips in the STO group.

**Results:**

Postoperative Harris hip scores, implant position, and the surgery time did not differ between the treatment groups. There were no differences in preoperative leg length discrepancy LLD (*P* = 0.46) and postoperative LLD (*P* = 0.56) between the two groups. Bone union occurred within 6 months after surgery in 12 hips in the GTT group (92.3%) and in 9 hips (81.8%) in the STO group. One case in the GTT group and two cases in the STO group had nonunion, and additionally, there was one case of postoperative nerve injury in the STO group, while no symptoms of nerve damage were observed in the GTT group.

**Conclusion:**

The GTT method demonstrated many advantages and reliable clinical results for Crowe type IV DDH patients undergoing THA. This is a surgical method that warrants further development and promotion clinically.

## Background

Crowe type IV developmental dysplasia of the hip (DDH) has the most complex degree of DDH lesions, which often causes earlier onset of hip pain in patients and the need for hip replacement surgery. These patients face several problems and challenges during total hip arthroplasty (THA), which aims at obtaining normal or close to normal hip biomechanics, prevent the abnormal increase in hip load, reduce the chance of wear and aseptic loosening, and prolong the life of the artificial hip prosthesis [1, 2]. Femoral shortening osteotomy is vital to the surgery. The most widely used surgery is subtrochanteric osteotomy, which has some potential complications such as nonunion and instability at the osteotomy site [3]. Some clinicians have used greater trochanter osteotomy, but there are few studies and reports related to this, mainly because of the limitations of bone nonunion [4]. In this study, we describe the use of a combination of greater trochanter osteotomy and tension wire fixation (GTT) in THA, which demonstrated good clinical results.

## Materials and methods

### General information

We selected patients who underwent THA surgery due to Crowe type IV DDH complicated with hip osteoarthritis in our hospital from January 2016 to July 2020. In the GTT group, senior surgeons treated 15 hips (13 patients) with a combination of trochanteric osteotomy and tension wire fixation. In the STO group, another set of senior surgeons treated 12 hips (11 patients) with subtrochanteric osteotomy. There were two patients in the GTT group and one patient in the STO group who had bilateral Crowe type IV DDH and underwent bilateral hip surgery in two separate operations. No cases were lost to follow-up. At the final follow-up, clinical scores and radiographic results were available for 14 hips in the GTT group and 12 hips in the STO group. A female patient in the GTT group was not included in the final analysis as she underwent THA following a fall. All patients included in this study signed informed consent, and all research procedures were in accordance with the WMA Declaration of Helsinki and approved by the Institutional Ethics and Review Committee. Clinical characteristics of the patients are shown in Table 1.

**Table 1:** The comprision of demographics and clinical results of the patients in the two groups

Variable	GTT group (*n* = 13)	STO group (*n* = 11)	*P* value
Female (*n*)	10	9	ns
Unilateral/bilateral hip dislocation (*n*)	11/2	10/1	ns
Age (range, year)	43.8 (29–54)	42.8 (28–52)	0.76
Body mass index (range, kg/m^2^)	24.6 (17.2–29.5)	22.8 (17.4–28.7)	0.25
LLD befor operation (range, cm)	5.7 (4.1–7.3)	5.4 (3.9–6.6)	0.46
LLD after operation (range, cm)	0.7 (0.1–1.5)	0.8 (0.3–1.6)	0.56
The time of operation (range, min)	115.1 (92–132)	121.7 (95–146)	0.17
Harris hip scores (range, points)			
Baseline	36.0 (20–53)	36.6 (27–56)	0.86
1 month	56.9 (47–64)	54.6 (40–67)	0.48
3 month	70.4 (60–80)	68.4 (51–82)	0.54
6 month	82.4 (71–90)	78.4 (60–88)	0.17
12 month	86.4 (75–93)	82.3 (63–93)	0.17
The final follow-up	88.1 (80–93)	84.3 (63–95)	0.16

*GTT* grochanteric osteotomy combined with tension wire fixation, *STO* subtrochanteric osteotomy, *LLD* leg length discrepancy, *HHS of baseline* HHS before surgery, *ns* no significant difference

### Preoperative plan

**Fig. 1:**
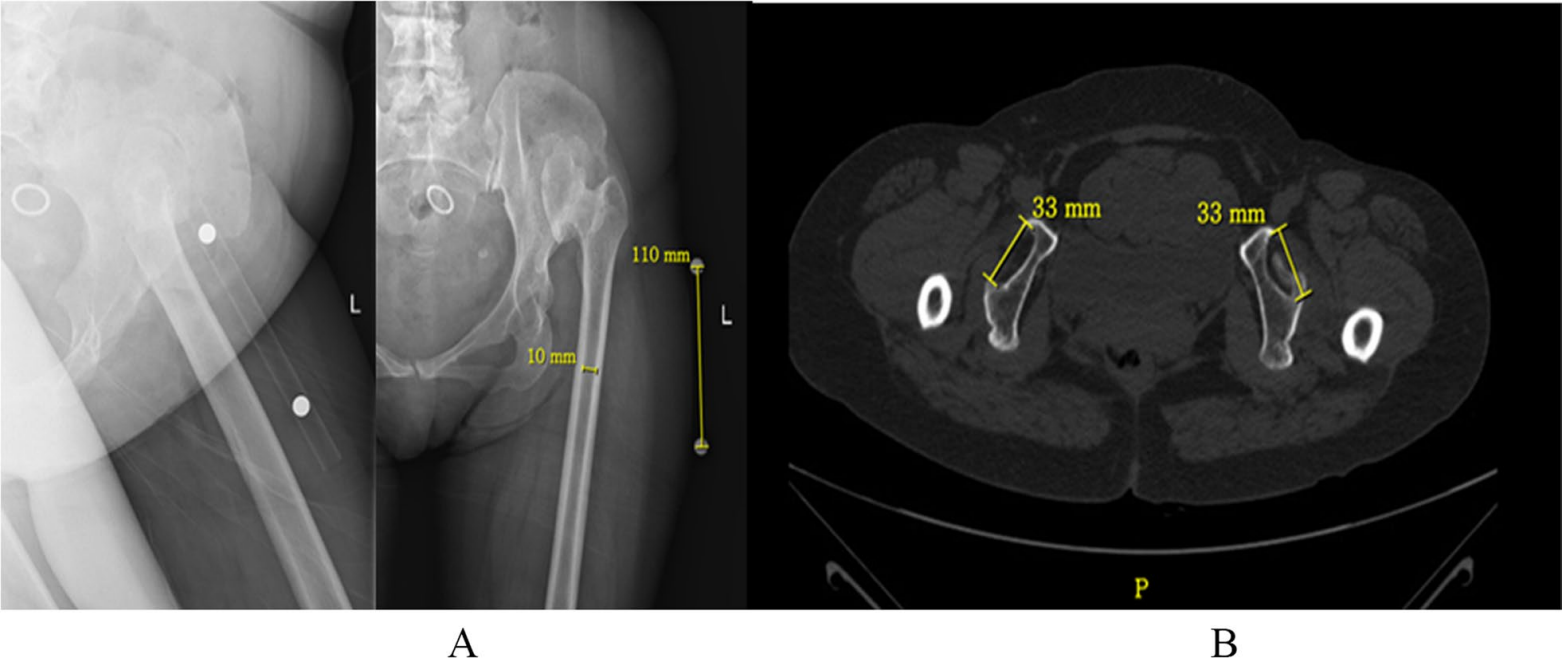
Preoperative measurements were performed using X-ray and CT scans. **A** We stuck a 100 mm-long marker rod with metal balls on both sides on the outside of the thigh, and took an X-ray of the femur. Measuring the diameter of the medullary cavity at the narrowest part of femoral diameter on X-ray film and calculating the actual diameter of the femoral medullary cavity can avoid the error caused by X-ray magnification and provide the basis for the types of femoral prosthesis that may be used during operation. Photos with marker posts were added. **B** Preoperative CT scanning of the hip joint was performed to evaluate the position and diameter of the true acetabulum and to guide the selection of acetabular prosthesis size and reconstruction of the true acetabulum

Before the surgery, X-ray and CT scan investigations are essential to evaluate and decide the selection of prosthesis (Fig. 1). In the standing position, we measured the distance between the anterior superior iliac spine and the medial malleolus and calculated the preoperative leg length discrepancy (LLD). At the same time, we assessed the preoperative Harris hip scale (HHS) scores for patients as the baseline HHS.

### Surgical procedure

**Fig. 2:**
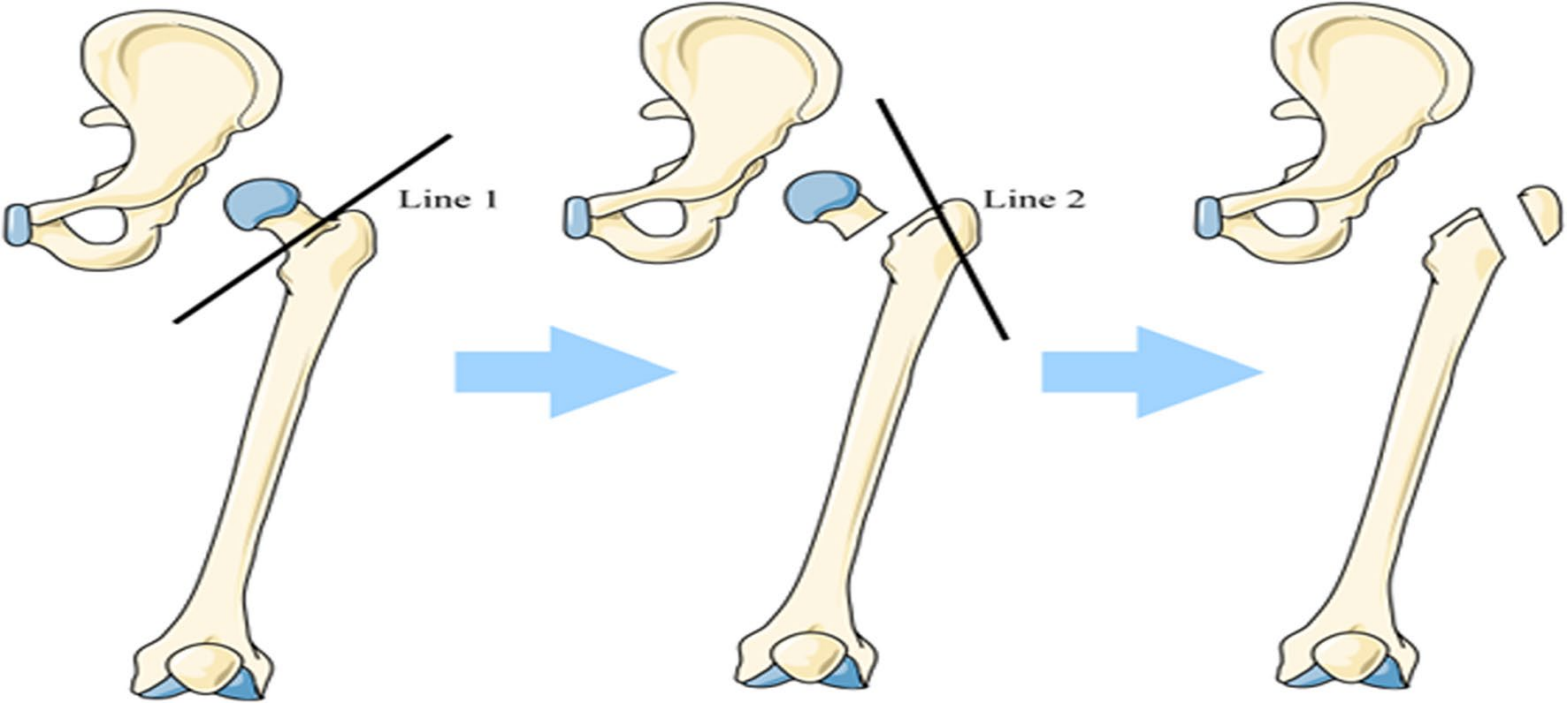
The first step is to amputate the femoral neck (Line 1), and the second step is to perform oblique osteotomy of the greater trochanter (Line 2) along the inner side of the greater trochanter and the upper edge of the femoral neck to the greater trochanter and the lateral femoral cortical bone, forming the soft tissue cuff of the gluteus medius and lateral femoral muscles, ensuring that the amputated greater trochanter bone has a good blood supply, and then the force arm of the gluteus medius muscle can be adjusted as needed when the greater trochanter is fixed

**Fig. 3:**
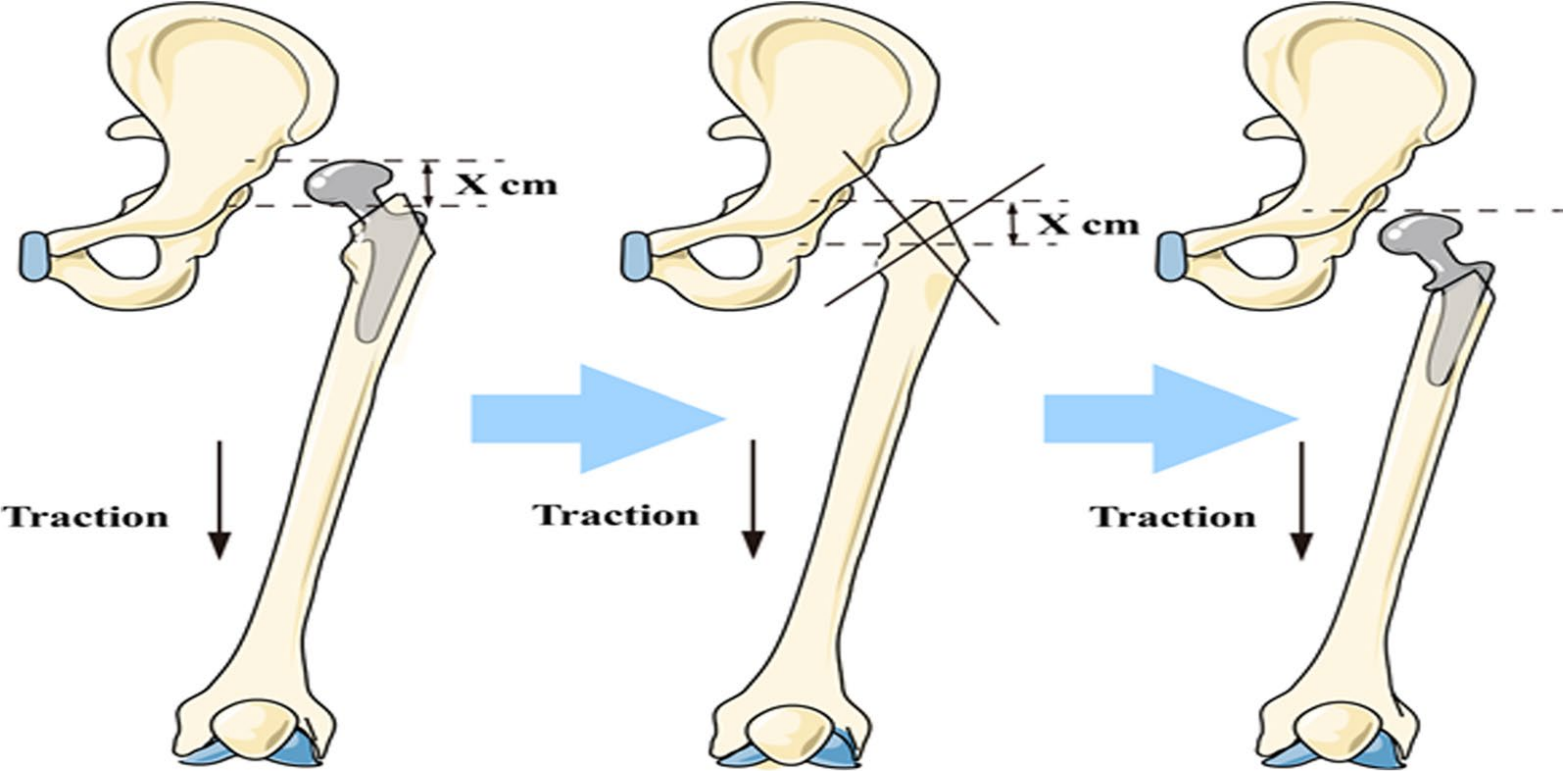
We enlarged the femoral bone marrow cavity to reach the maximum contact with the femoral cortex, inserted the test model, pulled the lower limb to try to restore, ensuring that there is no tension in the sciatic nerve, measured the length of the femur that needs to be shortened when it can be restored, and cut off the proximal femur according to this length. We put the femoral prosthesis in the “press-fit” mode and paid attention to ensure the correct anteversion

**Fig. 4:**
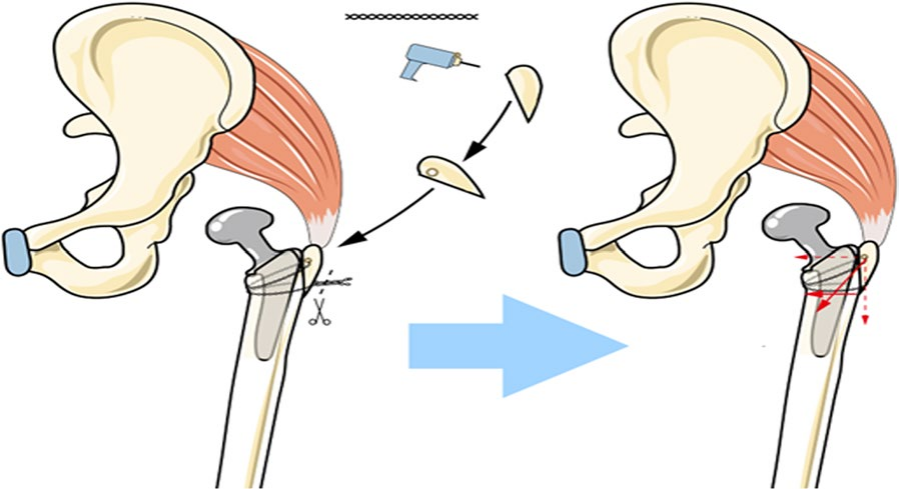
We used a 3.0 mm drill bit to drill a bone path transversely at the thickest part of the bone block, crossed the intertwined double-stranded steel wire through the bone path, crossed and bypassed the two ends of the steel wire from the inner side of the femur to the outer side of the femur respectively, and tightened the two ends of the steel wire at the outer side of the lower edge of the greater trochanter bone block and the femoral transition, in the same direction as the previous double-stranded steel wire winding, and the bone block was firmly fixed to the proximal femur. The steel wire at the proximal end tightened the greater trochanter inward and downward. The steel wire at the lower and outer sides of the greater trochanter bone block tightened the bone block inward in the lower direction. At the same time, it avoided the tendency of the distal end of the bone block to shift outward due to the tightening of the steel wire at the proximal end, which was similar to the fixation of the tension band

In the GTT group, the femoral neck was amputated during the operation, and at the same time, the oblique osteotomy of the greater trochanter was performed (Fig. 2). The greater trochanter could be easily opened after osteotomy to facilitate the exposure of the true mortar. We pulled the amputated greater trochanter and the sleeve back to accurately locate the true mortar according to the direction of the joint capsule and the “steps” at the transition between the true mortar and the pelvis. We took care to protect the acetabulum. The metal acetabular prosthesis was implanted with the “press fit” method and fixed with 2–3 screws. Next the management of the femoral side was treated (Fig. 3). The Wagner cone taper handle was used to adjust the femoral lateral anteversion according to the specific position of the femur. As the femoral medullary cavity of patients with Crowe type IV DDH is very thin, even the smallest femoral prosthesis prepared before the operation may still be too large. For these patients, we made a controlled longitudinal split of the proximal femur during the operation, and steel wire was used preemptively to prevent iatrogenic fracture so that the femoral prosthesis could be smoothly placed and firmly fixed. We reset the hip joint, adjusted the thickness of the greater trochanter bone block with a swing saw on the osteotomy surface of the greater trochanter for shaping, adjusted the height of the greater trochanter bone block to be fixed and offset it according to the specific situation of the soft tissue tension, firmly fixed the greater trochanter bone block with the soft tissue cuff of the gluteus medius muscle and lateral femoral muscle on the outside of the proximal femur with steel wire, and accurately reconstructed the eccentricity of the greater trochanter to ensure good gluteus medius muscle tension and blood supply of the bone block and obtain a stable hip and facilitate bone healing. Our fixation method of the greater trochanter bone block steel wire was consistent with the principle of biomechanics (Fig. 4).

In the STO group, a transverse femoral osteotomy was performed about 2–3 cm distal to the tip of the lesser trochanter. The amount of subtrochanteric shortening required was decided intraoperatively based on the degree of overlap between the proximal and distal femoral segments.

### Statistical analysis

All statistical analyses were performed using SPSS version 22.0 (SPSS Inc., Chicago, IL, USA). Quantitative variables are expressed as mean and standard deviation with range.

Independent samples and *t*-tests were used to assess differences of the quantitative data between the treatment groups. Categorical variables were compared between the 2 study groups using Fisher exact test. *P* value of < 0.5 was considered statistically significant.

### Source of funding

The authors received no external funding for this study.

## Results

### Clinical results

**Fig. 5:**
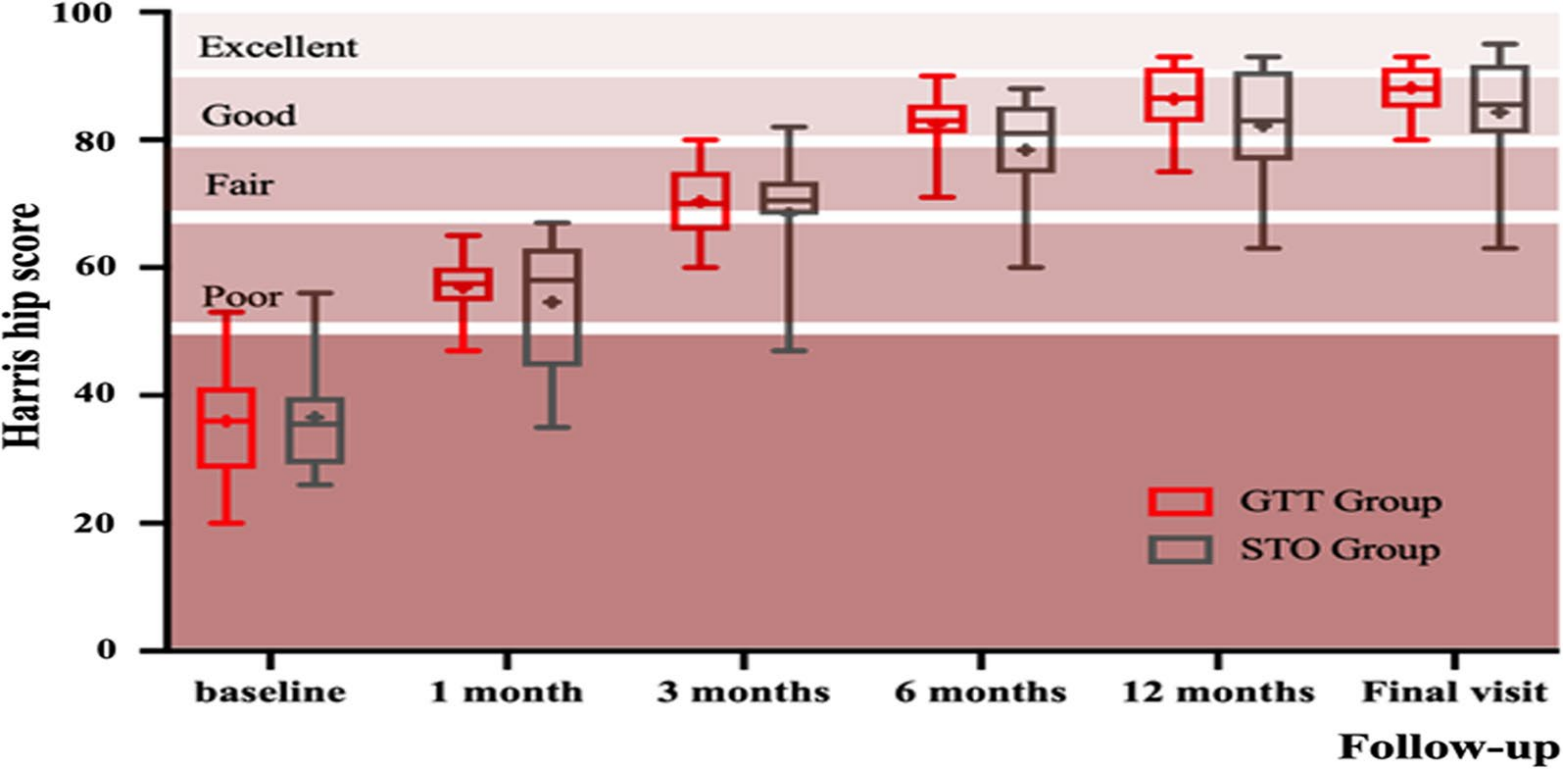
Temporal trends in Harris Hip Scale scores after surgery. Data are mean (95% confidence interval)

Harris hip scale (HHS) scores did not differ between the two groups (Table 1). Temporal trends for the HHS scores (Fig. 5) showed that hip function gradually improved from baseline to 6 months after surgery and was reasonably stable about 6 months later.

**Fig. 6:**
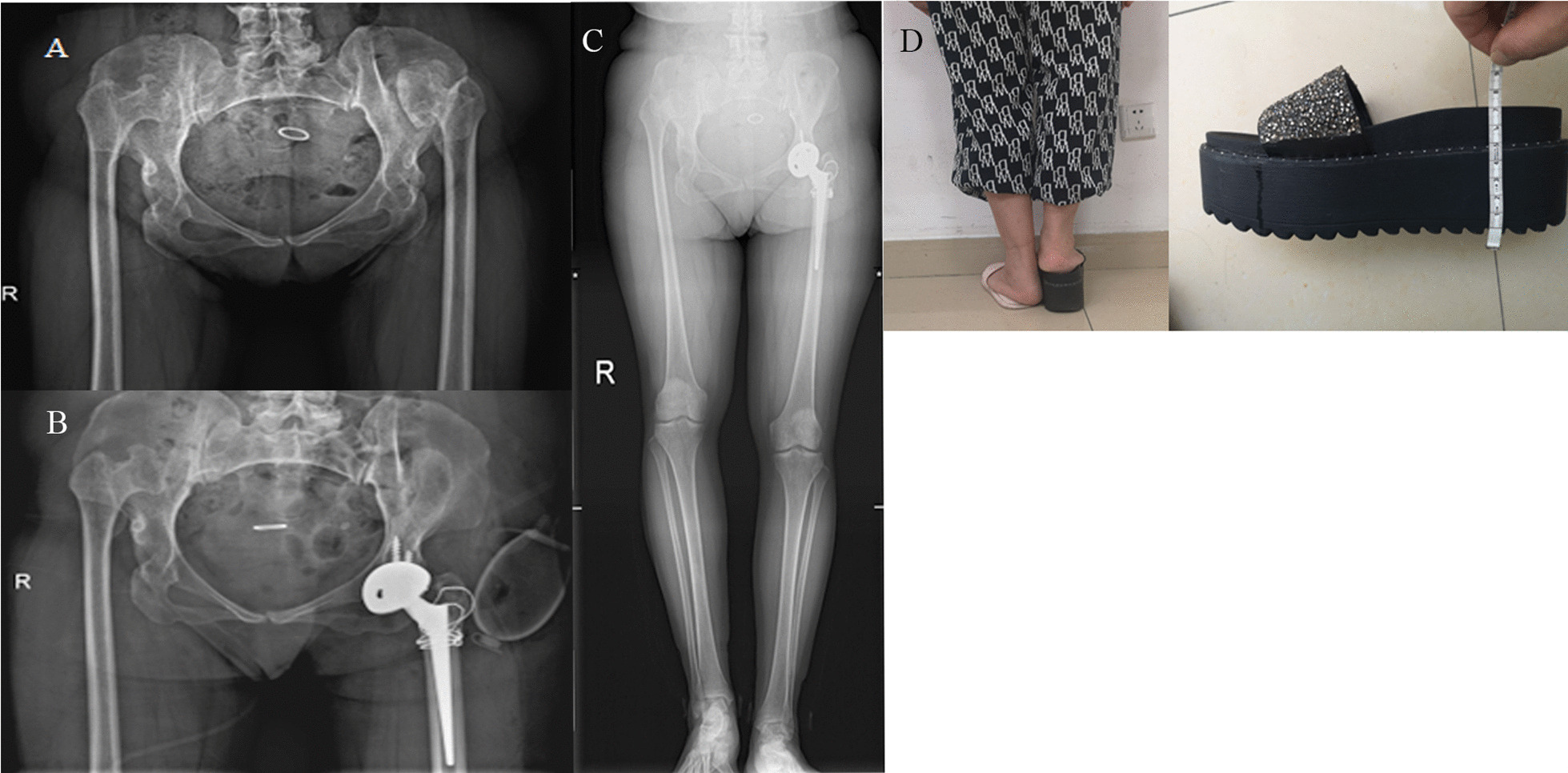
Stage-wise details of a 49-year-old female patient with bilateral Crowe type IV DDH who underwent THA surgery. **A** Preoperative X-ray exams of bilateral hip joints showed that both femoral heads were deformed and detached from the outside of the true mortar, which was shallow and small; **B** and** C** In the first stage, the left THA was performed first. The postoperative X-ray showed that the left acetabular prosthesis was in the true acetabulum, and the greater trochanter bone block was well fixed with steel wire; **D** Three months after the first surgery, the right limb was significantly shorter than the left, about 7.5 cm

**Fig. 7:**
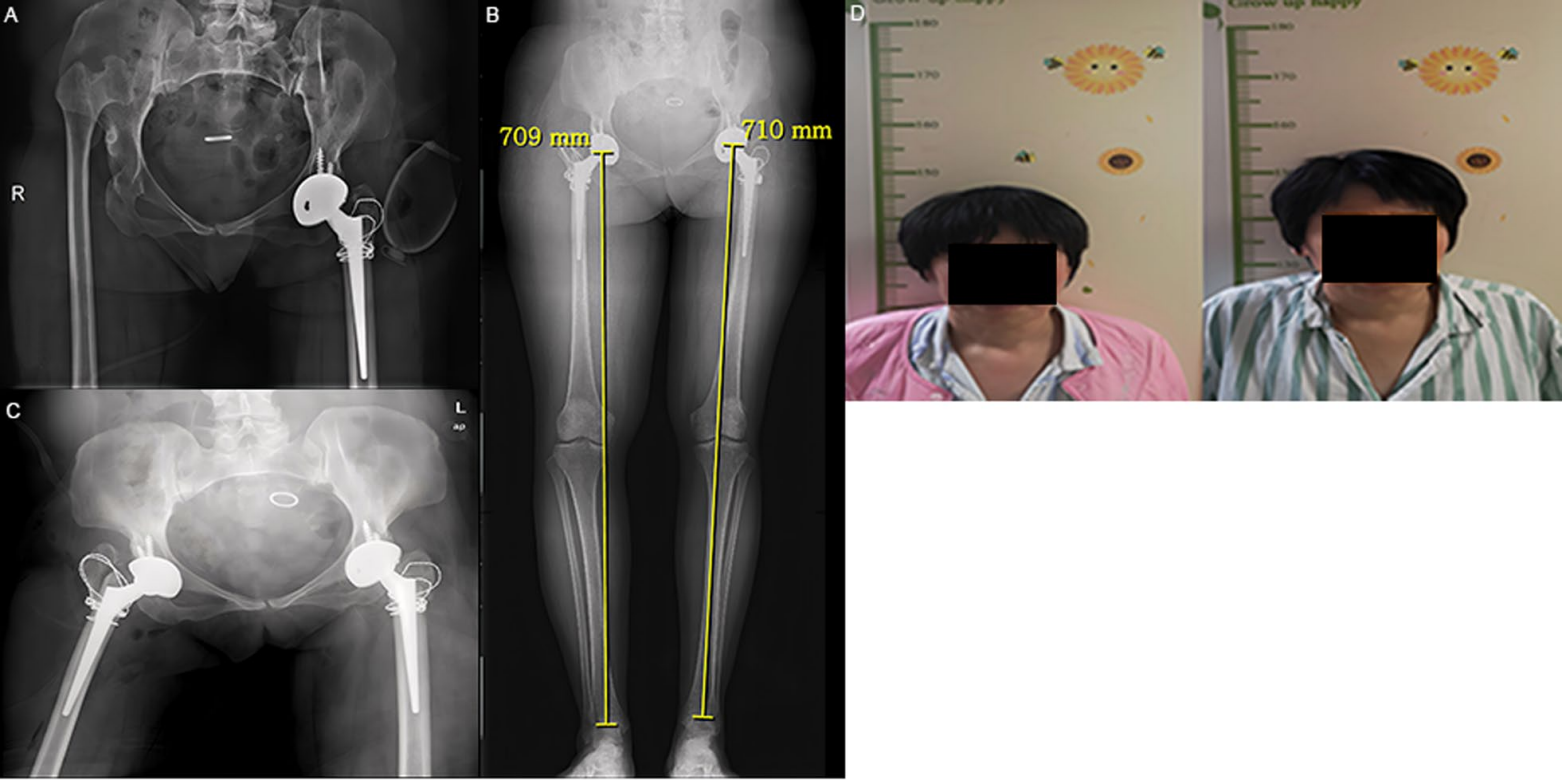
Three months later, the preoperative X-ray of the right THA was performed at the second stage. **A** The X-ray image after the first operation; **B** On the third day after the second stage of right THA; **C** The full-length X-ray of both lower limbs in a standing position showed that the length difference of both limbs was 0.1 cm; **D** The height was measured on the third day after the second operation. Compared with that before the operation, the height was about 7.5 cm longer, and the LLD was 0.1 cm

Both, the preoperative LLD and the postoperative LLD did not differ between the two groups (Table 1). One patient with a bilateral surgery in the GTT group had the same length of both lower limbs before the operation. Both lower limbs were lengthened by about 7.5 cm after the surgery, and the LLD was 0.1 cm (Figs. 6 and 7). This patient did well after the surgery and recovered smoothly.

### Radiographic results

There were no differences associated with the implant position between the two groups (Table 2).

**Table 2:** The comprision of radiographic data of the patients in the two groups

Variable	GTT group (*n* = 13)	STO group (*N* = 11)	*P* value
Mean cup orientation (range)			
Inclination	48.0° (39.3°–57.5°)	46.5° (38.5°-55.2°)	0.34
Anteversion	18.2° (10.2°–23.8°)	17.6° (12.5°-22.8°)	0.68
Stem alignment within − 3° to 3° (*n*)	13	11	ns
The time of bone healing (month)	3.4 (2.6–4.0)	4.3 (3.0–5.1)	0.32
Nonunion	1/13	2/11	0.58

*GTT* grochanteric osteotomy combined with tension wire fixation, *STO* subtrochanteric osteotomy, *ns* no significant difference

One case in the GTT group and two cases in the STO group showed bone nonunion 6 months after the surgery according to the postoperative X-ray (*P* = 0.58). However, the patient in the GTT group had near-normal joint function, whereas patients in the STO group had lower limb pain and limited mobility.

### Complications

In addition to the nonunion cases mentioned above, one patient had symptoms of nerve injury in the STO group, but there were none in the GTT group (*P* = 0.46). One patient in the GTT group, a 49-year-old female, had an accidental fall 2 months after the surgery, resulting in dislocation of the hip joint. The closed reduction was unsuccessful, and open exploration was performed. During the operation, we found dislocation of the femoral head and formation of the original callus at the osteotomy of the greater trochanter. For the dislocated femoral head, the soft tissue attached to the well-fixed greater trochanter and with good tension, confined the reduction of the femoral head. The femoral head was not repositioned until removal of the steel wire holding the greater trochanter, and removal of the greater trochanter bone block and associated soft tissue. Then according to the soft tissue tension, the greater trochanter block was reattached to the proximal femur after refreshing the osteotomy surface.

No complications such as dislocation, nonunion, periprosthetic fracture, nerve injury, deep vein thrombosis, infection, or heterotopic ossification was observed in other patients.

## Discussion

There are many pathological changes and structural abnormalities in patients with Crowe type IV DDH [1, 5, 6], and there are many problems and challenges in the treatment of these patients.

For surgery in Crowe type IV DDH patients, sufficient exposure and extensive soft tissue release are required. The commonly used exposure methods are the posterolateral approach combined with a subtrochanteric osteotomy, and there are two ways: one is exposure after subtrochanteric osteotomy [7], and the other is subtrochanteric osteotomy after acetabular treatment [8]. In the former method, the proximal femur has a large bone mass and surrounding soft tissue, so the room for pulling is limited. The latter method is not helpful for the exposure of the acetabulum, but only for shortening the femur. In GTT, the first step is to amputate the femoral neck, and the second step is to perform oblique osteotomy of the greater trochanter along the inner side of the greater trochanter and the upper edge of the femoral neck to the greater trochanter and the lateral femoral cortical bone. Compared to the proximal portion of the femur after subtrochanteric osteotomy, the greater trochanteric bone block, which is only attached to the soft tissue sleeve of the gluteus medius and vastus lateralis muscles, is smaller, and there is greater room to pull the greater trochanteric bone block apart—this is more convenient to expose the real acetabulum that is not easy to locate. Furthermore, based on years of clinical experience, we also found the lesser trochanter tends to remain approximately horizontal with the true acetabulum. So, the preserved lesser trochanter can help locate the true acetabulum. These characteristics are helpful for the exposure in the surgery.

Many subtrochanteric shortening osteotomy methods are reported, including transverse, oblique, Z-shaped, and double V-shaped methods. These have no significant difference in the stability of the osteotomy site [9]. Transverse STO has some potential complications, such as nonunion and instability at the osteotomy site due to the limited contact area of the osteotomy and the lack of sufficient rotational stability [3]. In addition, the diameters of the distal and proximal ends of the subtrochanteric shortening osteotomy are different, resulting in a mismatch between the prosthesis matching the distal medullary cavity and the proximal medullary cavity and the instability of the proximal end of the osteotomy. Furthermore, the subtrochanteric osteotomy site is from cortical bone to cortical bone, which is not conducive for bone healing. All of this increases the possibility of bone nonunion [10].

Other STOs have similar rates of nonunion as transverse osteotomies [11]. In GTT, the excess part of the proximal femur was directly removed, and the femoral prosthesis was directly inserted into the distal medullary cavity, and this can effectively avoid the mismatch between the proximal medullary cavity and the prosthesis. Furthermore, the greater trochanter bone block has many cancellous bone components, with attachment to the soft tissue sleeve of the gluteus medius muscle, lateral femoral muscle, and good blood supply. In addition, our steel wire fixation method could provide firm fixation of the bone block. These are conducive to bone healing at the osteotomy site.

Few relevant clinical studies have focused on the use of greater trochanteric osteotomy in hip arthroplasty in Crowe type IV DDH patients. Jin et al. performed hip arthroplasty in 13 patients with Crowe type IV DDH. They first cut the proximal femur horizontally at the middle level of the lesser trochanter, then cut the proximal femur longitudinally from the apex of the greater trochanter to form the inner and outer parts, and the inner part was removed [12]. This method is also conducive to expose the acetabulum and for the adjustment of fixation position of the proximal lateral part according to the needs of the offset. However, it is not adjusted according to the actual length at the time of the reduction, and this may have the risk of too much or too little osteotomy. A steel plate was used to fix the bone block, which does not conform to the biomechanical principle of this fixation and increases the risk of trauma and the cost of treatment. Due to the femoral prosthesis, only steel wire can be used for plate fixation instead of screw fixation, and the fixation effect is impacted. Chen et al. used the method of proximal femur shaping for Hartofilakidis Type C DDH hip replacement [13]. This method has a relatively large range of osteotomy, leading to no protection of soft tissue blood supply at the distal end of the bone block, which may affect bone healing. In addition, the method of fixation with steel wire could not balance the tension of the gluteus medius muscle. By comparison, the osteotomy bone block with GTT was smaller and composed of cancellous bone and there was no need to peel off the proximal femur. The soft tissue sleeves of the gluteus medius and lateral femoral muscles of the bone block were completely attached, and the blood supply was relatively good, which was conducive to bone healing. Additionally, the steel wire fixation method in GTT can effectively balance the tension of the gluteus medius muscle by pulling the bone block downward and inward when tightening the steel wire, which conforms to the mechanical principle of this fixation. Lee et al. used steel wire to fix the greater trochanter bone block in hip replacement for femoral intertrochanteric fracture. The method of fixation and mechanical principles are different from GTT [14].

The reconstruction of the true acetabulum and reduction of femoral head are crucial [3]. Direct reduction, facing the obvious hypertrophy and hyperplasia of the joint capsule, extensive contracture of the surrounding muscles and the soft tissues caused by long-term dislocation, especially when the limb is extended by more than 4 cm, may cause injury to the sciatic nerves. At present, this issue is solved by inferior trochanteric osteotomy or extensive soft tissue release [1, 15]. For STO, the reduction was performed only after the femoral osteotomy was completed and the prosthesis was installed, at which time there was no opportunity for soft tissue compensation, resulting in sciatic nerve injury due to excessive traction during the reduction process. For GTT, the proximal femur that needs to be shortened was cut off according to the tension of surrounding soft tissue and after ensuring that there is no tension in the sciatic nerve. The femoral prosthesis was installed for reduction before the greater trochanter was fixed. As the greater trochanter bone block was fixed after reduction, it could be easily restored without the limitation of the abductor, and the possibility of injury to the sciatic nerve was very small. In the GTT group, there was one patient who underwent a bilateral surgery. Both lower limbs were lengthened by about 7.5 cm after the operation, and no sciatic nerve injury was observed (Figs. 6 and 7); 7.5 cm is much longer than the 4 cm reported in the literature, which shows the potential advantage of this method to avoid nerve injury.

The reconstruction of femoral offset in hip arthroplasty is of great significance. It can provide an appropriate abductor arm, avoid abductor weakness or tightness, ensure the stability of the hip joint, reduce joint wear, avoid hip joint impact, and maintain a good gait [16, 17]. Due to the long-term dislocation of the femoral head, the abductor movement in Crowe IV DDH patients changes significantly, becoming shorter and contracted, so that the force arm is in a pathological state [6]. After subtrochanteric osteotomy in Crowe type IV DDH patients, the rotation alignment of proximal bone block and distal femur is adjusted to obtain 10°–15° femoral prosthesis anteversion and optimal distal and proximal alignment, to ensure good eccentricity and joint stability [15]. Since the femoral stem enters the distal femoral medullary cavity through the medullary cavity of the proximal bone block, and the far and near ends are completely aligned, the reconstruction of the eccentricity is determined when the prosthesis is placed, and the femoral eccentricity cannot be further adjusted. In GTT, after the reduction of the hip joint, the greater trochanter bone block was shaped according to the specific situation of abductor muscle tension. After finding the most appropriate fixation height and offset of the greater trochanter bone block (through the control of the thickness of the greater trochanter bone block), it was fixed on the outside of the proximal femur with steel wire for the accurate reconstruction of the greater trochanter eccentricity and to ensure a good abductor arm and obtain a stable hip joint.

There are some limitations to this retrospective study. The sample size was small and the follow-up time was short, which could not fully explain the results of this method. The movement direction of the gluteus medius muscle was changed during the surgery, and the arm of the abductor muscle was reconstructed, but the change of gluteus medius muscle strength was not observed post-surgery. We did not observe and analyze the long-term survival rate of the prosthesis, which is required for future technical improvements. Two different groups of doctors performed the two kinds of surgery, which may have biased the measurement.

## Conclusions

The GTT method is a reliable solution for patients with Crowe type IV DDH, with demonstrated potential advantages in surgical exposure, soft tissue release, avoidance of nerve injury, and biomechanical reconstruction.

## Data Availability

The datasets generated and/or analysed during the current study are not publicly available due to the lack of an online platform but are available from the corresponding author on reasonable request.

## Abbreviations

DDH: Developmental dysplasia of the hip
THA: Total hip arthroplasty
HHS: Harris hip scale
